# Molecular Imaging of p53 in Mouse Models of Cancer Using a Radiolabeled Antibody TAT Conjugate with SPECT

**DOI:** 10.2967/jnumed.124.267736

**Published:** 2024-10

**Authors:** Hudson Alakonya, Sofia Koustoulidou, Samantha L. Hopkins, Mathew Veal, Javier Ajenjo, Deborah Sneddon, Gemma Dias, Michael Mosley, Julia Baguña Torres, Francesca Amoroso, Amanda Anderson, Alison H. Banham, Bart Cornelissen

**Affiliations:** 1 Department of Oncology, Oxford Institute for Radiation Oncology, University of Oxford, Oxford, United Kingdom;; 2 Department of Chemistry, University of Oxford, Oxford, United Kingdom;; 3 Nuffield Division of Clinical Laboratory Sciences, Radcliffe Department of Medicine, University of Oxford, Oxford, United Kingdom; and; 4 Nuclear Medicine and Molecular Imaging, University Medical Centre Groningen, University of Groningen, Groningen, The Netherlands

**Keywords:** molecular imaging, p53, cancer, pancreatic ductal adenocarcinoma

## Abstract

Mutations of p53 protein occur in over half of all cancers, with profound effects on tumor biology. We present the first—to our knowledge—method for noninvasive visualization of p53 in tumor tissue in vivo, using SPECT, in 3 different models of cancer. **Methods:** Anti-p53 monoclonal antibodies were conjugated to the cell-penetrating transactivator of transcription (TAT) peptide and a metal ion chelator and then radiolabeled with ^111^In to allow SPECT imaging. ^111^In-anti-p53-TAT conjugates were retained longer in cells overexpressing p53-specific than non–p53-specific ^111^In-mIgG (mouse IgG from murine plasma)-TAT controls, but not in null p53 cells. **Results:** In vivo SPECT imaging showed enhanced uptake of ^111^In-anti-p53-TAT, versus ^111^In-mIgG-TAT, in high-expression p53^R175H^ and medium-expression wild-type p53 but not in null p53 tumor xenografts. The results were confirmed in mice bearing genetically engineered KPC mouse–derived pancreatic ductal adenocarcinoma tumors. Imaging with ^111^In-anti-p53-TAT was possible in KPC mice bearing spontaneous p53^R172H^ pancreatic ductal adenocarcinoma tumors. **Conclusion:** We demonstrate the feasibility of noninvasive in vivo molecular imaging of p53 in tumor tissue using a radiolabeled TAT-modified monoclonal antibody.

## Visual Abstract

**Figure:**
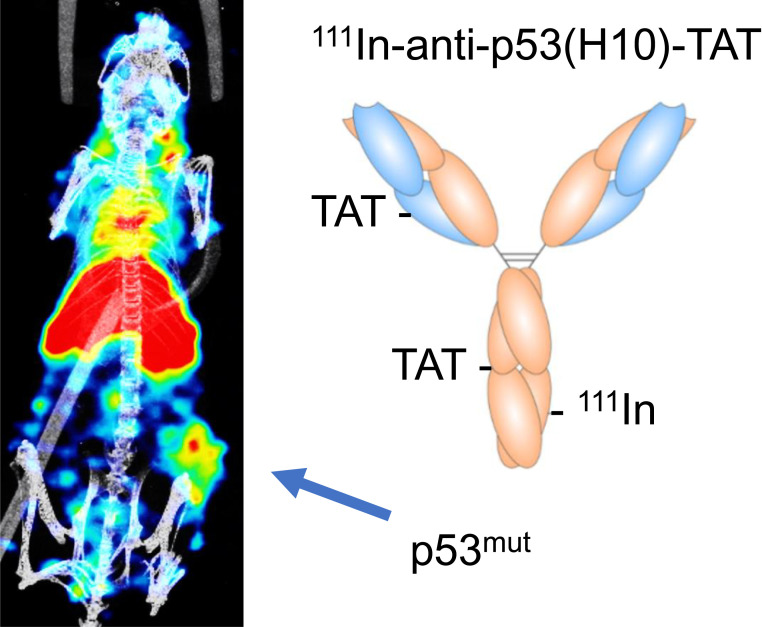

The p53 protein is one of the most extensively studied proteins in cancer research. Mutation of its encoding gene, *TP53,* can result in the expression of mutant dysfunctional protein (1*,*^2^). *TP53* is mutated in more than half of all cancers. Mutant p53 (p53^mut^) proteins gain new capabilities including dominant negative effects over the remaining wild-type p53 (p53^wt^), wild-type p63, and wild-type p73 tumor suppressor proteins, as well as gain-of-function effects promoting cancer aggressiveness and resistance to therapies (3). Under normal conditions, p53^wt^ has a relatively short half-life because it is constantly targeted for proteasomal degradation by its main negative regulator, MDM2, through an autoregulatory negative feedback loop (4). Conversely, many p53 mutant forms escape degradation, leading to accumulation of p53 in those cancer cells (5). Most p53 mutations correspond to a single amino acid substitution occurring commonly within the central DNA binding domain. Six amino acids are most frequently mutated (residues 45, 248, 273, 282, 220, and 175, mostly in the DNA binding domain of the protein). These so-called hot-spot mutations account for 30% of all p53 mutations in human cancers (6), with higher penetrance in ovarian serous carcinoma (96%), small cell lung cancer (85%), and pancreatic ductal adenocarcinoma (PDAC, 75%) (2*,*^6^*,*^7^). p53^mut^ overexpression in cancers has been shown to occur in preinvasive lesions and invasive tumors, as well as in PDAC, and is associated with poor prognosis (8), altered immune interactions, and differential response to treatment. Therefore, a molecular imaging probe capable of detecting p53 overexpression could positively impact early cancer diagnosis and provide useful information for treatment stratification and evaluation.

There are presently no noninvasive methods for directly detecting p53 in vivo. Current p53-based detection techniques are based on ex vivo immunohistochemistry and flow cytometry using anti-p53 antibodies or on genetic sequencing of biopsied samples (9). All available methods are invasive and insufficient for distant, deep-seated, or occult metastases and do not account for tumor heterogeneity. Molecular imaging with a labeled agent targeting p53 can overcome this challenge. To allow imaging of p53 using techniques allowing whole-body imaging such as PET or SPECT, several obstacles must be addressed. First, p53 mutants are numerous and diverse. More than a thousand p53 mutations have been documented in cancer, classified as either structural, that is, inducing conformational changes (e.g., R175H, R249S, and R282W); contact mutations, which disrupt p53’s ability to interact with its consensus sequences (R248W and R273W); and null mutations, resulting in an absence of p53 protein (10*,*^11^). An effective p53 imaging agent must therefore bind to a variety of p53 proteins (wild type, contact mutant, and structural mutant).

More challenging still, p53 is an intracellular protein, accumulating in the cell nucleus when mutated, which necessitates an imaging agent possessing cellular penetration and nuclear localization potential. Despite their excellent selectivity and affinity, the large size and hydrophilic nature of monoclonal antibodies (mAbs) hinders them from binding nuclear proteins. This limitation can be overcome by conjugating mAbs to a cell-penetrating peptide capable of translocating a mAb cargo across both the cell and the nuclear membranes, that is, with a nuclear localization sequence (12). The HIV-1–derived transactivator-of-transcription (TAT) peptide (GRKKRRQRRRPPQGYG, nuclear localization sequence underlined) has been successfully used, by us and others, to deliver radiolabeled mAbs for PET and SPECT imaging of intranuclear epitopes including phosphorylated histone protein γH2AX, p21, and p27 (12–22).

Here, we conjugated the TAT peptide to an anti-p53 mAb developed in-house, 49A1/H10 (H10), or the commercial anti-p53 antibody 1C12 and radiolabeled it with ^111^In, resulting in ^111^In-anti-p53-TAT. These radiolabeled compounds bind specifically to total p53 (wild type as well as mutated, murine as well as human) and accumulate in tumors expressing or overexpressing p53. This work describes the first—to the best of our knowledge—imaging agent for noninvasive imaging of p53 in vivo. This study demonstrates a preclinical strategy for molecular imaging of p53, with potential for further exploration and clinical translation.

## MATERIALS AND METHODS

Full details are presented in the supplemental materials (available at http://jnm.snmjournals.org).

### Cell Culture

A panel of cancer cell lines with varying p53 status and expression was characterized for p53 expression using Western blot, flow cytometry, and immunohistochemistry (Supplemental Fig. 1): human cancer cell lines MiaPaCa-2, HT1080, H1299, PANC-1, FAMPAC-1, BxPC-3, and AsPC-1. KPC mouse (23*,*^24^)–derived cell line B8484 originated from LSL-Kras^G12D/+^, LSL-Trp53^R172H/+^, Pdx-1-Cre (KPC) mice (25). P2FIR cells were derived from LSL-Kras^G12D/+^, LSL-Trp53^flox^, Pdx-1-Cre mice (25) and were used as negative controls (full methods in the supplemental materials). Protein levels were compared with normal human pancreatic tissue lysate, obtained commercially (Novus Biologicals).

### Anti-p53 H10 Generation and Characterization

Mouse anti-p53 antibody clone H10 was generated by immunization of female BALB/c mice with KLH-conjugated peptide amino acids 13–27 of human p53 (PLSQETFSDLWKLLP), with high analogy to murine p53 (PLSQETFSGLWKLLP). This section of the protein is not commonly mutated. Antibodies were characterized using Western blot, pulldown assay, flow cytometry, and immunocytochemistry. Full details are presented in the supplemental materials.

### Conjugation and Radiolabeling

Conjugation of TAT peptide and diethylenetriaminepentaacetic acid (DTPA) chelator to H10, and subsequent radiolabeling with ^111^In, were performed using strain-promoted azide alkaline click chemistry using TAT-N_3_ and N_3_-Bn-DTPA after dibenzocyclooctyne conjugation of the antibody, as previously described (19), resulting in ^111^In-H10-TAT. Mouse IgG from murine plasma (mIgG), conjugated and radiolabeled in the same way, was used as a nonspecific control in these studies, resulting in ^111^In-mIgG-TAT.

In a separate set of studies, using KPC mouse–derived murine PDAC cell lines, allografts, and KPC animals, a commercial anti-p53 antibody (1C12, catalog no. 2524; Cell Signaling) was used. p-SCN-BnDTPA (to allow radiolabeling with ^111^In) and TAT conjugation (to enable cellular penetration and nuclear localization) were conjugated to the antibody by 1-ethyl-3-[3-dimethylaminopropyl]carbodiimide hydrochloride/*N*-hydroxysuccinimide chemistry as described previously (16), resulting in ^111^In-1C12-TAT. Isotype-matched murine IgG (mIgG) was used as a nonspecific control in KPC mouse and KPC allograft studies.

The ability of ^111^In-H10-TAT to be blocked from binding to its target by native, unmodified H10 was compared with the nonradiolabeled DTPA-H10-TAT. FAMPAC-1 cells in a 48-well plate (1 × 10^5^ cells per well) were fixed with 4% (w/v) paraformaldehyde for 10 min, washed, and permeabilized with 1% (v/v) Triton X-100 (Dow Chemical Co.) in phosphate-buffered saline for 20 min, after which nonspecific binding was blocked with 250 μL of radioimmunoassay blocking buffer (phosphate-buffered saline plus 2% bovine serum albumin [bovine serum albumin plus 0.1% polysorbate-20]) for 1 h. The cells were then exposed to 2 nM ^111^In-H10-TAT, together with increasing concentrations of either native H10 or DTPA-H10-TAT, for 1 h (total volume, 500 μL). The wells were washed and lysed with ice-cold 0.1 M NaOH, and the amount of ^111^In associated with the cells in each well was determined by automated γ-counting (Wizard^2^ 2480; Perkin Elmer). Similar tests were performed using ^111^In-1C12-TAT.

### In Vitro Uptake and Retention

Aliquots of 1 × 10^5^ MiaPaCa-2, HT1080, and H1299 cells in 24-well plates were incubated for 1 h with 1.5 nM ^111^In-H10-TAT or ^111^In-mIgG-TAT (1 MBq/μg). The nonbound, membrane-bound, and internalized radioimmunoconjugates were collected, and the amount of ^111^In associated with each fraction was determined. Retention of ^111^In in cells, after incubation of cells with either ^111^In-H10-TAT or ^111^In-mIgG-TAT for 1 h and refreshing of the medium, was determined after various intervals, as previously described (16). The same study was performed using ^111^In-1C12-TAT in B8484 versus P2FIR cells.

### Animal Models

All animal experiments were performed according to the U.K. Animals (Scientific Procedures) Act of 1986 and with local ethical committee approval. All animals were housed in individually ventilated cages in groups of up to 5, in an artificial day–night cycle facility with ad libitum access to food and water.

Immunodeficient mice bearing MiaPaCa-2, HT1080, or H1299 cell tumor xenografts on the right flank, or KPC mouse–derived B8484 or P2FIR xenografts, were used for in vivo imaging studies. KPC mice spontaneously generate PDAC lesions because of a pancreas-specific *KRAS*^G12D^ and *TP53*^R172H^ mutation, under Pdx-1-Cre control (26).

### In Vivo Imaging

^111^In-H10-TAT or ^111^In-mIgG-TAT (5 MBq/5 μg) in phosphate-buffered saline was administered to mice via the lateral tail vein. SPECT/CT images were acquired using a VECTor^4^CT scanner (MILabs) at 24, 48, and 72 h after injection as described previously (27). Allograft-bearing and non–allograft-bearing KPC mice were injected intravenously with ^111^In-1C12-TAT or ^111^In-mIgG-TAT isotype control (5 MBq/5 μg) in phosphate-buffered saline and imaged 24, 48, and 72 h later. [^18^F]FDG was used to detect murine tumors in KPC mice. The supplemental materials provide full details.

### Ex Vivo Biodistribution Studies

After image acquisition at 72 h, the mice were culled and samples of blood, selected normal tissues, and tumor were collected. The weight and amount of radioactivity were determined using an automated γ-counter (HiDex). The percentage injected activity per gram of tissue was calculated. Immediately after the tumor radioactivity had been counted, samples were taken and processed for Western blot analysis; the remaining tissues were flash-frozen and stored at −80% for sectioning, autoradiography, and histologic analysis. Full details are in the supplemental materials.

### Statistical Analysis

Prism version 9 or higher (GraphPad Software) was used for statistical analysis of all data and for curve fitting. One-way ANOVA with the Tukey post hoc test was used to compare differences in group means for p53 protein levels in different cell lines. All experiments were performed at least in triplicate and presented as mean ± SD unless otherwise stated.

## RESULTS

### Monoclonal Anti-p53 Antibody H10 Binds Selectively to Total p53 with Nanomolar Affinity

p53 was expressed at varying levels in human PDAC cells (Supplemental Figs. 1A–1H). We evaluated FAMPAC-1 (high levels of p53^R175H^), MiaPaCa-1 (high levels of p53^R248W^), HT1080 (moderate levels of p53^wt^), and H1299 (no p53, null p53 [p53^null^]), as well as murine PDAC B8484 (high levels of p53^R172H^) and P2FIR (no p53, p53^null^), by Western blot, flow cytometry, and enzyme-linked immunosorbent assay, with commercially available p53 antibody (PAb 10442-1-AP or 1C12) or kits.

For molecular imaging of p53, we used 2 different antibodies to demonstrate the modularity of the approach: 1C12 and H10. Despite the availability of ubiquitous anti-p53 antibodies, we developed our own, given the relatively large amounts of antibody needed for molecular imaging and the associated high cost. Some of the commercially available anti-p53 antibodies showed, in our hands, large batch-to-batch variation. A proprietary anti-p53 mouse mAb clone was produced in-house by immunization of mice using a C-terminal fragment of human p53, highly analogous to murine p53. From a selection of hybridoma lines (Supplemental Figs. 2A and 2B), clone H10 was selected on the basis of its differential binding to human and murine p53 mutant and wild-type–expressing cells and cell lysates versus p53^null^ cells. Other clones also showed these characteristics, but H10 hybridoma cells proved more resistant to fetal bovine serum–free growing medium. H10 was shown to bind to p53^wt^ and p53^mut^ in whole-cell lysates in human and murine cells expressing large amounts of p53 (FAMPAC-1, MiaPaCa-1, B8484) or medium amounts of p53 (HT1080) but not in p53^null^ H1299 or P2FIR cells (Supplemental Fig. 2C). The results were confirmed by pulldown assay, flow cytometry, and immunocytochemistry (Supplemental Fig. 2D). To additionally assess the binding selectivity of H10, an immune-pulldown assay was performed using biotinylated H10, which showed only p53 staining in the pulldown fraction (Supplemental Fig. 2D). Immunofluorescence microscopy and a flow cytometry–based saturation binding study, using a panel of cell lines, showed that H10 binds its target, whether it be p53^wt^, p53^R175H^, or p53^R248W^, with better than nanomolar affinity (dissociation constant < 1 nM) (Supplemental Figs. 2E–2G). We confirmed that the commercially available anti-p53 mouse mAb 1C12 binds human and murine p53, as validated using murine PDAC B8484 (p53^mut^) and P2FIR (p53^null^) cells using Western blot, flow cytometry, and immunocytochemistry (Supplemental Figs. 3A–3C). The binding affinity (dissociation constant) of 1C12 was 9.0 ± 2.2 nM, as determined by enzyme-linked immunosorbent assay (Supplemental Fig. 3D).

### Conjugation and Radiolabeling of Anti-p53 Does Not Affect Affinity

**FIGURE 1.**
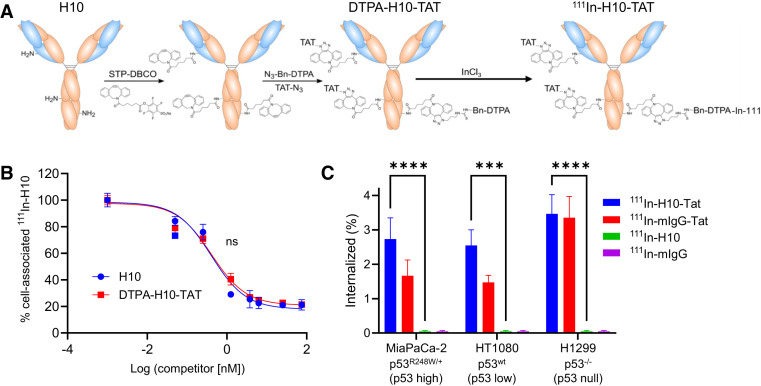
(A) ^111^In-H10-TAT was synthesized by conjugation of activated 4-sulfo-2,3,5,6-tetrafluorophenyl ester of dibenzocyclooctyne to mAb, after which click chemistry was used to conjugate azide-DTPA to allow radiolabeling with ^111^In, and azide-TAT peptide for cell penetration. Labeling with ^111^InCl_3_ allows detection through its γ-ray emissions and molecular SPECT imaging. (B) Comparison of native H10 with conjugated DTPA-H10-TAT’s ability to block binding of ^111^In-H10 to p53 in fixed and permeabilized p53^R175H^-overexpressing FAMPAC cells showed no significant differences. (C) Uptake of ^111^In-H10-TAT was no different from that of ^111^In-mIgG-TAT in 3 different cell lines but far higher than non–TAT-conjugated versions. ***P < 0.001. ****P < 0.0001. DBCO = dibenzocyclooctyne; STP = 4-sulfo-2,3,5,6-tetrafluorophenyl.

Anti-p53 antibody H10 was modified with dibenzocyclooctyne on lysine moieties, using an activated ester to allow strain-promoted azide–alkyne cycloaddition with the TAT peptide (GRKKRRQRRRPPQGYG-hA(N_3_)) and using the metal ion chelator N_3_-Bn-DTPA to allow radiolabeling with ^111^In (Fig. 1A). A dibenzocyclooctyne:IgG ratio of 5.0 ± 0.2 was achieved. On average, 1.6 TAT moieties and 3.3 DTPA moieties were conjugated to H10 and mIgG alike. Instant thin-layer chromatography showed a 98% and 99% radiochemical yield and purity for ^111^In-mIgG-TAT and ^111^In-H10-TAT, respectively (Supplemental Fig. 4). ^111^In-1C12-TAT was synthesized, using a different method, as described before (16). Conjugation with TAT and DTPA did not significantly affect the p53 binding affinity of H10 or 1C12. The ability of the native, unmodified, antibody to displace a radiolabeled version (without TAT) in p53-expressing cells was not significantly different from that of DTPA-anti-p53-TAT (half-maximal inhibitory concentration, 0.39 ± 0.01 vs. 0.43 ± 0.03 nM to displace ^111^In-H10, respectively; *P* > 0.05) (Fig. 1B). DTPA-H10-TAT still bound p53 in lysates from FAMPAC-1 and HT1080 cells, but not H1299, as well as nonmodified H10, in Western blot assays (Supplemental Fig. 2H). The results were similar for ^111^In-1C12-TAT (Supplemental Fig. 5). None of the conjugates resulted in any significant cell toxicity in vitro at relevant concentrations as demonstrated by 3-(4,5-dimethylthiazol-2-yl)-2,5-diphenyltetrazolium bromide, clonogenic, or resazurin assays (Supplemental Fig. 6).

### In Vitro Uptake and Retention of ^111^In-Anti-p53-TAT Correlates with p53 Expression

**FIGURE 2.**
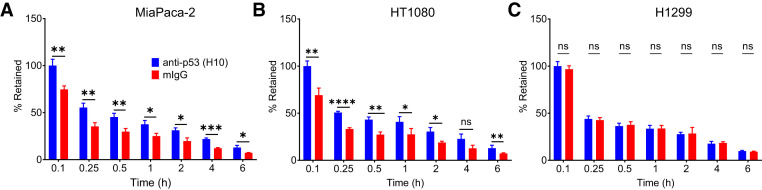
After initial 1-h exposure of cells and washing, retention of ^111^In-H10-TAT was significantly higher than that of ^111^In-mIgG-TAT in p53^mut^-overexpressing MiaPaCa-2 cells (A) and in p53^wt^ HT1080 cells (B) but not in p53^null^ H1299 cells (C). *P < 0.05. **P < 0.01. ***P < 0.001. ****P < 0.0001. ns = not statistically significant.

No difference was observed in the uptake of ^111^In-H10-TAT versus ^111^In-mIgG-TAT in a panel of cell lines after a 1-h exposure (*P* > 0.05; Fig. 1C). Both conjugates showed far higher uptake than did non-TAT–conjugated antibodies (*P* < 0.001; Fig. 1C). However, in a pulse-and-chase study, the amount of ^111^In-H10-TAT retained in p53^mut^ MiaPaCa-2 and p53^wt^ HT1080 cells was significantly higher than the amount of ^111^In-mIgG-TAT retained (*P* < 0.05), but this was not so in p53^null^ H1299 cells (*P* < 0.05; Fig. 2). The same was true for ^111^In-1C12-TAT in p53^mut^ B8484 cells, where it was retained longer than was ^111^In-mIgG-TAT, but this was not so in p53^null^ P2FIR cells (*P* < 0.01; Supplemental Fig. 5E).

### ^111^In-H10-TAT Enables In Vivo Imaging of p53 in Mouse Models of Cancer

**FIGURE 3.**
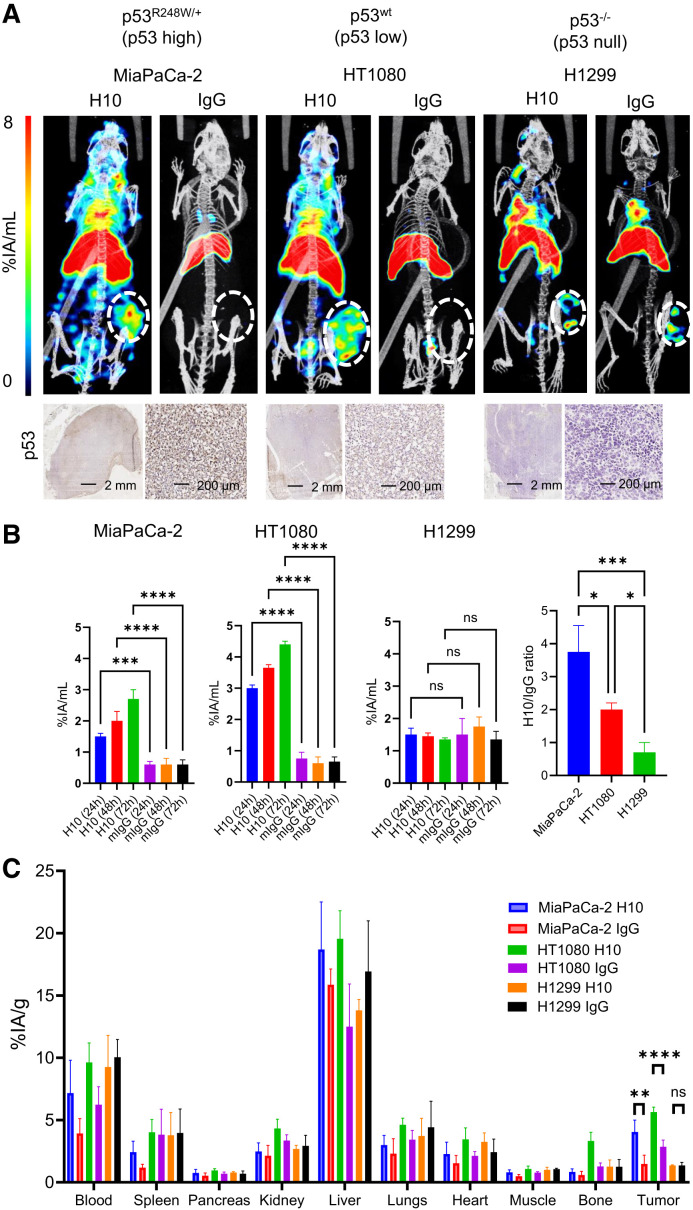
(A) SPECT/CT imaging 72 h after intravenous administration of ^111^In-H10-TAT or ^111^In-mIgG-TAT (5 MBq, 5 μg) in BALB/c *nu/nu* mice bearing p53^mut^ MiaPaCa-2, p53^wt^ HT1080, or p53^null^ H1299 xenografts. At bottom, immunohistochemistry staining for p53 in tissue harvested from tumor xenografts confirms p53 expression levels. (B–E) Volume-of-interest analysis of tumor uptake over time of ^111^In-H10-TAT or ^111^In-mIgG-TAT in mice bearing MiaPaCa-2, HT1080, or H1299 xenografts. Tumor uptake ratio of ^111^In-H10-TAT or ^111^In-mIgG-TAT is in line with p53 expression in tumor xenografts. (F) Full biodistribution data 72 h after intravenous administration of ^111^In-H10-TAT or ^111^In-mIgG-TAT shows differences only in tumor xenografts, not in normal tissue. *P < 0.05. **P < 0.01. ***P < 0.001. ****P < 0.0001. %IA = percentage injected activity; ns = not statistically significant.

In vivo imaging in mice bearing MiaPaCa-2 (p53^R175H^ high expression) and HT1080 (p53^wt^ medium expression) xenografts after administration of ^111^In-H10-TAT (5 μg, 5 MBq) showed higher tumor uptake of ^111^In-H10-TAT than of ^111^In-mIgG-TAT, but this was not so in mice bearing p53^null^ H1299 xenografts (Figs. 3A–3E). In both MiaPaCa-2 and HT1080 tumors, but not in H1299 tumors, a steady increase in the amount of H10-associated radioactivity was observed over time (*P* < 0.05), as well as an increase in H10/mIgG uptake ratios (Figs. 3B–3D). Uptake of labeled mIgG-TAT remained stable and relatively low across all time points (Fig. 3B). Ex vivo biodistribution 72 h after intravenous administration showed equally increased tumor uptake of ^111^In-H10-TAT in MiaPaCa-2 (*P* < 0.01) and HT1080 xenografts (*P* < 0.001), compared with ^111^In-mIgG-TAT, but not in H1299 xenografts (*P* = 0.15) (Fig. 3F). No significant differences were observed in any of the normal tissues we evaluated (*P* > 0.05). ^111^In-H10-TAT versus ^111^In-mIgG-TAT ratios across the various tumors were in line with p53 levels (high, modest, and null in MiaPaCa-2, HT1080, and H1299, respectively) as measured by immunohistochemistry. Full data are presented in Supplemental Figures 7–9.

### ^111^In-1C12-TAT Allows p53 Imaging in KPC Allograft Tumors

**FIGURE 4.**
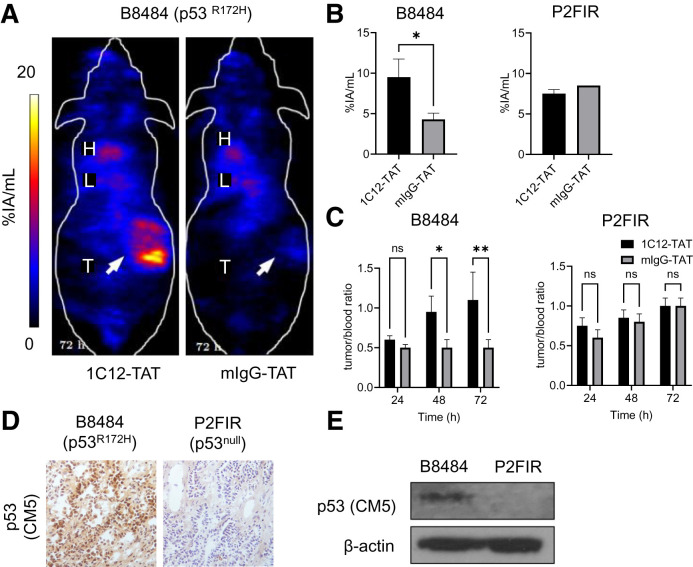
(A) SPECT/CT imaging 72 h after intravenous administration of ^111^In-1C12-TAT vs. ^111^In-mIgG-TAT (5 MBq, 5 μg) in mice bearing B8484 murine PDAC allografts shows uptake of former but not latter. (B and C) Volume-of-interest analysis of tumor uptake, and tumor-to-blood ratios of ^111^In-1C12-TAT or ^111^In-mIgG-TAT in mice bearing p53^R172H^ B8484 or p53^null^ P2FIR allografts, show significant uptake in former, not latter. (D and E) Immunochemistry and Western blot analysis of p53 expression in tumor allograft tissue using rabbit polyclonal anti-p53 antibody CM5 confirmed p53 expression. *P < 0.05. **P < 0.01. %IA = percentage injected activity; H = heart; L = liver; ns = not statistically significant; T = tumor.

B8484 (p53^R172H^) murine allografts demonstrated significantly higher uptake of ^111^In-1C12-TAT than of ^111^In-mIgG-TAT (*P* < 0.05), measured by in vivo imaging and ex vivo biodistribution at 72 h after injection (Fig. 4; Supplemental Fig. 10). The tumor-to-blood ratios increased over time for ^111^In-1C12-TAT but not for ^111^In-mIgG-TAT (Fig. 4C). No significant differences between ^111^In-1C12-TAT and ^111^In-mIgG-TAT uptake were observed in p53^null^ P2FIR allografts. p53 expression, high in B8484 and absent in P2FIR allografts, was confirmed by immunohistochemistry and Western blot (Figs. 4D and 4E).

### ^111^In-1C12-TAT Allows Tumor Imaging in KPC GEM Mice with Spontaneous PDAC Tumors

The biodistribution of ^111^In-1C12-TAT was assessed in genetically engineered KPC mice, a more accurate model of human PDAC, exhibiting desmoplasia that may affect tumor uptake. PDAC tumors were detected by MRI and [^18^F]FDG PET in KPC mice from 20 wk old onward. Uptake of ^111^In-1C12-TAT in non–tumor-bearing mice was similar to that in BALB/c *nu/nu* mice (Supplemental Fig. 11). Diffuse uptake of ^111^In-1C12-TAT was present across the body, most evidently in the bladder, the heart, and the facial papillomas that are common in KPC mice. In tumor-bearing KPC mice, uptake of ^111^In across the whole pancreas/tumor tissue was measured as 2.4 ± 1.1 percentage injected activity per gram of tissue. Colocalization of focal uptake of ^111^In-1C12-TAT in the pancreas autoradiography and hematoxylin and eosin staining, indicating PanIN-3 lesions and invasive PDAC foci, was also seen (Supplemental Fig. 11F).

## DISCUSSION

p53 is the most frequently mutated protein among all cancers. p53 mutants are overexpressed in many cancers, including colorectal, pancreatic, esophageal, and non–small cell lung cancer, and are associated with cancer aggressiveness, chemoresistance, and overall poor prognosis. In PDAC, p53^mut^ overexpression is a hallmark of most invasive pancreatic intraepithelial neoplasia lesions (PanIN-3) and invasive tumors. Most p53 mutations in PDAC are missense, occurring mainly in the DNA binding domain of p53, leading to overexpressed p53. Koorstra et al. showed that p53 overexpression is pronounced in PanIN-3 lesions and in invasive PDAC but not in normal pancreatic tissues or noninvasive lesions (8). Recently, p53^mut^ overexpression has been the target of innovative therapeutic strategies aiming to restore native structure to ensure degradation and rescue from dominant negative effects (1*,*^2^). Thus, visualization of p53 expression would allow patient selection for these novel therapies (9), as well as patient stratification and prediction and evaluation of the relative efficacies of conventional chemotherapy. Currently, determining p53 status is based mostly on DNA sequencing or immunohistologic assessment in a biopsy. To the best of our knowledge, no in vivo imaging method exists to allow this. Molecular imaging using PET or SPECT will allow quantitative, noninvasive, repeatable whole-body assessment of p53 expression.

We previously explored how antibodies, which do not cross the cell membrane, can be modified with the TAT peptide and radiolabeled to allow PET or SPECT imaging and can successfully target intracellular and even intranuclear protein epitopes (18*,*^28^–^30^). Here, we used non–site-specific modification of the IgG, using click chemistry or 1-ethyl-3-[3-dimethylaminopropyl]carbodiimide hydrochloride/*N*-hydroxysuccinimide conjugation chemistry. The particular click approach used here allowed us to readily control and quantify antibody modification. Neither conjugation method led to marked modification of the antibody’s affinity for its target epitope, nor did targeting of p53 with the conjugates result in measurable toxicity. We opted to radiolabel using the γ-emitting radionuclide ^111^In to allow SPECT imaging. In animal studies, SPECT provides better spatial resolution than PET. Previously, we showed that a similar conjugate labeled with ^89^Zr for PET imaging resulted in similar biodistribution (17). For any human applications, labeling with ^89^Zr combined with PET imaging would be preferred, given the superior resolution and sensitivity of PET over SPECT in human-sized scanners.

This method allowed for imaging of p53 in MiaPaCa-2 and HT1080 subcutaneous tumors, displaying significant tumor uptake and excellent specificity compared with a nonspecific control based on a nonspecific mIgG. Although the uptake of ^111^In-H10-TAT itself was markedly higher than that of IgG control in both MiaPaCa-2 and HT1080 xenografts, this did not correlate linearly with p53 levels. Differences in the enhanced-permeability-and-retention effect between tumors, including HT1080, explain this (31). Thus, the ratio of tumor uptake between ^111^In-H10-TAT and ^111^In-mIgG-TAT was considered, which correlated well with p53 expression.

To further explore the applicability of p53 imaging with ^111^In-anti-p53-TAT conjugates, we looked at KPC PDAC allograft–bearing mice and performed a case study on a set of KPC mice with or without a spontaneous PDAC tumor. Here, we used a commercially available murine anti-p53 mAb (1C12) as the basis of the IgG-TAT conjugate. In KPC mice, although tumor uptake was lower, possibly because of the pronounced desmoplasia around the tumor tissue, uptake of ^111^In-1C12-TAT was observed by SPECT imaging. High uptake correlated with areas of PanIN-3 lesions and invasive PDAC tissue, as demonstrated by autoradiography, thus—together with the above—suggesting PDAC-associated p53 targeting in KPC mice.

Our study demonstrated the possibility of noninvasive in vivo imaging of p53, upregulated because of mutation, using radiolabeled mAbs.

We did not look here at a large range of p53 mutants, merely at a single representative from one of the major categories of p53^mut^. However, the antibody H10 was raised against a C-terminal region that is not commonly mutated, thus targeting total p53 protein, and binding to structural and contact mutants was confirmed. Of course, the anti-p53-TAT conjugates cannot be used to visualize TP53 mutations that result in no expression of the p53^null^ variants. We did not explore modulation of p53^wt^.

Detection of p53 expression will depend on the copy number per cell. We previously showed that the lowest detection threshold of an artificial intranuclear GFP epitope with anti-GFP-TAT conjugates was approximately 0.25 × 10^6^ copies per cell (19). Thus, minimal p53^mut^ expression levels that allow mAb visualization are needed, depending on antibody affinity, among other parameters. The p53 mutants used here are overexpressed, making it a more straightforward target than the DNA double-strand-break marker γH2AX, which accumulates and dissipates over relatively short times, measured in hours, contrary to the much slower kinetics of a radiolabeled antibody. An optimization of the IgG-TAT architecture we used here was described recently by Tietz et al., who used trimeric TAT peptide complexes (32). This may lead to higher uptake and superior signals in vivo and lower detection limits.

Taken together, we present the first—to our knowledge–proof of the concept of imaging p53 expression in vivo using molecular imaging with radiolabeled modified anti-p53 antibodies. We demonstrate the feasibility of imaging overexpressed p53 using a radiolabeled TAT-conjugated mAb. Molecular imaging of p53 in cancer may aid in early diagnosis, prognosis prediction, treatment selection, and treatment monitoring.

## DISCLOSURE

This research was supported by the Medical Research Council (Bart Cornelissen and Sofia Koustoulidou), by Cancer Research U.K. through the Oxford Institute for Radiation Oncology (Bart Cornelissen), by the Medical Research Council (through a DPhil fellowship to Sofia Koustoulidou), and by the Rhodes Trust (through a DPhil studentship to Hudson Alakonya). The funders had no role in the design, data collection, data analysis, and reporting of this study. Bart Cornelissen holds patents on radiopharmaceutical technology, not relating to the compound in this manuscript. Bart Cornelissen acted as a paid consultant for Theragnostics Ltd. and Blue Earth Diagnostics. No other potential conflict of interest relevant to this article was reported.

## Notes

KEY POINTS **QUESTION:** Can we image expression of p53, the most mutated protein in cancer? **PERTINENT FINDINGS:** In a panel of cancer cell lines with varying p53 status in xenograft tumor–bearing animals and in a genetically engineered model of spontaneous PDAC in mice, we demonstrated the possibility of imaging an accumulation of mutated p53. This is, to the best of our knowledge, the first time that this most important of proteins, which accumulates in the cancer cell nuclei, has been visualized using noninvasive molecular imaging. **IMPLICATIONS FOR PATIENT CARE:** After clinical translation, imaging and quantification of p53 may predict prognosis, aid therapy selection, and enable therapy evaluation.
